# IL22RA1/JAK/STAT Signaling Acts As a Cancer Target Through Pan-Cancer Analysis

**DOI:** 10.3389/fimmu.2022.915246

**Published:** 2022-07-08

**Authors:** Shuai Zhang, Guiyan Yang

**Affiliations:** ^1^ Department of Pathology and Laboratory Medicine, Davis Health, University of California, Sacramento, CA, United States; ^2^ College of Veterinary Medicine, Northeast Agricultural University, Harbin, China; ^3^ College of Veterinary Medicine, China Agricultural University, Beijing, China

**Keywords:** IL22RA1, immune response, jak-stat, overall survival, pan-cancer analysis

## Abstract

Cytokines and cytokine receptors are important mediators in immunity and cancer development. Interleukin 22 (IL22) is one of the most important cytokines which has protumor effect. Given that common and specific roles of cytokines/receptors in multiple cancers, we conducted a pan-cancer study to investigate the role of IL22RA1 in cancer using The Cancer Genome Atlas (TCGA) database. Notably, we found IL22RA1 transcript was upregulated in 11 cancer types compared with their corresponding control. The mRNA expression level of IL22RA1 was highest in the pancreas among tumor tissues. The higher expression of IL22RA1 was associated with worse overall survival rate in patients. A total of 30 IL22RA1-correlated genes (e.g. *IL17D*, *IL22RA2*, *IL20RB*, *IL10RA*, *IL10RB*, *TSLP* and *TYK2*) are involved in the JAK/STAT pathway which promotes tumor progression. The upregulation of IL22RA1 in tumors was correlated with immune cell infiltration level. Higher expression of IL22RA2, IL20RB, IL10RA, IL10RB, TSLP, TYK2, STAT1 and STAT3 was associated with decreased overall survival rate in patients. IL22RA1 mutation was observed more in uterine cancer and melanoma compared with the other cancer types. Deactivation of IL22RA1 induced a lot of changes in gene expression. IL22RA1 mutants had upregulated DNA damage/repair genes in uterine cancer, whereas downregulated genes in the FoxO signaling pathway. In melanoma, mutation of IL22RA1 can upregulate the HIF signaling pathway but downregulate metabolic pathways. Our study suggests that IL22RA1/JAK/STAT signaling can be an important target for cancer treatment.

## Introduction

Cancer has been a leading cause of death in the world since 2020 (World Health Organization). Among types of cancer treatment, immunotherapy is becoming one of the most effective strategies against cancer, as it can strengthen the power of the immune system (1). Nevertheless, immune responses play a dual role in cancer progression (2). It causes side effects and resistance which also influence the outcome of treatment. The cytokine-cytokine receptor interaction pathway is associated with cancer and other diseases (Comparative Toxicogenomics Database). However, the interaction mechanism is not fully understood. Although different cancers are molecularly distinct, many share common driver mutation and molecular signatures. It is vital to conduct a pan-cancer analysis of specific genes of interest and assess its correlation with clinical prognosis and potential molecular mechanisms.

IL22RA1 is an important heterodimeric receptor for interleukin 22 (IL22) which is involved in chronic inflammation and tumor development. IL22RA1 is expressed on cell membrane of nonimmune cells including epithelial cells from different organs or hepatocytes (3). Through binding to IL22RA1, IL22 activates the STAT3 pathway, thus regulating genes involved in cell proliferation and survival. As a mediator of immune cell-epithelial cell crosstalk, the association of IL22RA1 and immune cell infiltration in cancer is not clear.

IL22 promotes tumorigenicity in pancreatic ductal adenocarcinoma through IL22RA1/STAT3 signaling (4). IL22 signaling interacts with mutant KRAS to promote poor prognosis in colorectal cancer (5). In muscle invasive bladder cancer, the poor outcome is also associated with high expression of IL22RA1 (6). The expression of IL22 was low in glioblastoma and normal tissues, whereas IL22RA1 was increased in primary and recurrent glioblastomas compared with normal tissues (7). These studies indicate that IL22RA1 may be a target in cancer treatment.

Our study used the TCGA databases to conduct a pan-cancer analysis of IL22RA1 for the first time. Here the expression and mutation status of IL22RA1 as well as the associated genes and immune cells were covered.

## Methods

### TIMER database analysis

RNA seq data from TCGA database were performed using TIMER 2.0 (http://timer.comp-genomics.org/) (8) including IL22RA1 expression, gene-gene correlation, as well as association between IL22RA1 expression and infiltration levels of immune cells across 31 tumor types.

### Kaplan-Meier survival analysis

The overall survival rate of patients with high or low expression of target genes was performed using the TCGA/TARGET/GTEx RNA-Seq dataset based on UCSC Xena (https://xenabrowser.net).

### Protein interactions and pathway analysis

A total of 30 genes and proteins have been found related with IL22RA1 by NCI/Nature (http://www.ndexbio.org/). Pathway analysis of these 31 genes was performed by The Database for Annotation, Visualization and Integrated Discovery (DAVID) v6.8. The transcripts level for IL22RA1-related genes were analyzed using the TCGA/TARGET/GTEx RNA-Seq dataset through UCSC Xena.

### Mutation analysis

The effect of mutation status of IL22RA1 on gene expression in cancer was explored using TCGA database by muTarget (https://www.mutarget.com) (9) and UCSC Xena.

### Western blotting

Proteins were extracted from uterine cancer tissues and adjacent normal control tissues from dogs at Animal Hospital of Northeast Agricultural University. This study was approved by Ethical Committee of Northeast Agricultural University. Primary antibodies used were 1:1000 anti-IL22RA1 (Sigma-Aldrich, USA) and 1:7500 anti-β-actin (Bioss antibodies, China). Then membranes were incubated with secondary horseradish peroxidase-HRP conjugated antibodies (ZSGB-BIO, China). The protein bands were visualized using Champ Gel 6000 image system (Sage Creation, Beijing, China). Image-Pro Plus 6.0 software (Media Cybernetics, Washington, USA) was used to analyze the density for each band. Results are presented as the ratio of the intensity of the IL22RA1 band to the intensity of the β-actin band.

### Statistics

Data are expressed as means ± standard deviation (SD). Differences were considered statistically significant at *p* < 0.05. Differentially expressed genes were based on |log2 fold change| > 0.5 and adjusted *p* < 0.05. GraphPad Prism Version 8.0 (San Diego, CA, USA) was used for graph generation. All data were analyzed using one-way analysis of variance (ANOVA) followed by Tukey’s *post hoc* test.

## Results

### Expression of IL22RA1 based on pan-cancer analysis of TCGA data

**Figure 1:**
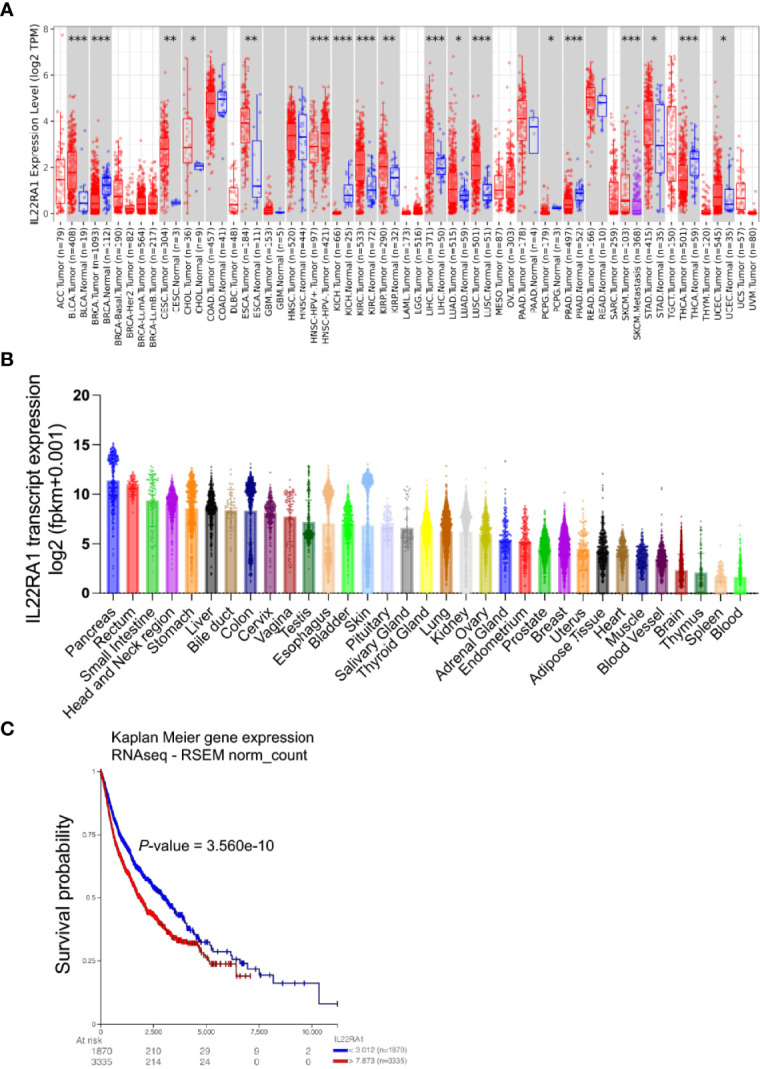
Expression of IL22RA1 is upregulated in tumors compared with normal tissues among 11 cancers and high expression worsens survival in patients. Data were obtained from TIMER2.0 (http://timer.cistrome.org) and TCGA/TARGET/GTEx databases. **(A)** Overexpression of *IL22RA1* transcript in multiple tumors. *p < 0.05, **p < 0.01, ***p < 0.001. **(B)** The transcriptive level of *IL22RA1* in different organs among diverse cancer types. **(C)** Kaplan Meier Plot shows the effect of higher *IL22RA1* expression on overall survival in patients.

Compared with normal tissues, the transcript level of IL22RA1 in tumor tissues was upregulated in a total of 11 cancers including bladder urothelial carcinoma (BLCA), cervical and endocervical cancer (CESC), cholangiocarcinoma (CHOL), esophageal carcinoma (ESCA), kidney renal clear cell carcinoma (KIRC), kidney renal papillary cell carcinoma (KIRP), liver hepatocellular carcinoma (LIHC), lung adenocarcinoma (LUAD), lung squamous cell carcinoma (LUSC), stomach adenocarcinoma (STAD), and uterine corpus endometrial carcinoma (UCEC) **(** **Figure 1A** **)**.

In humans, IL22RA1 was highly expressed in the pancreas, rectum, and small intestine **(** **Figure 1B** **)**. Notably, higher expression of IL22RA1 was associated with poor survival in patients **(** **Figure 1C** **)**.

### IL22RA1-related genes involve in the JAK/STAT signaling pathway and cancers

**Figure 2:**
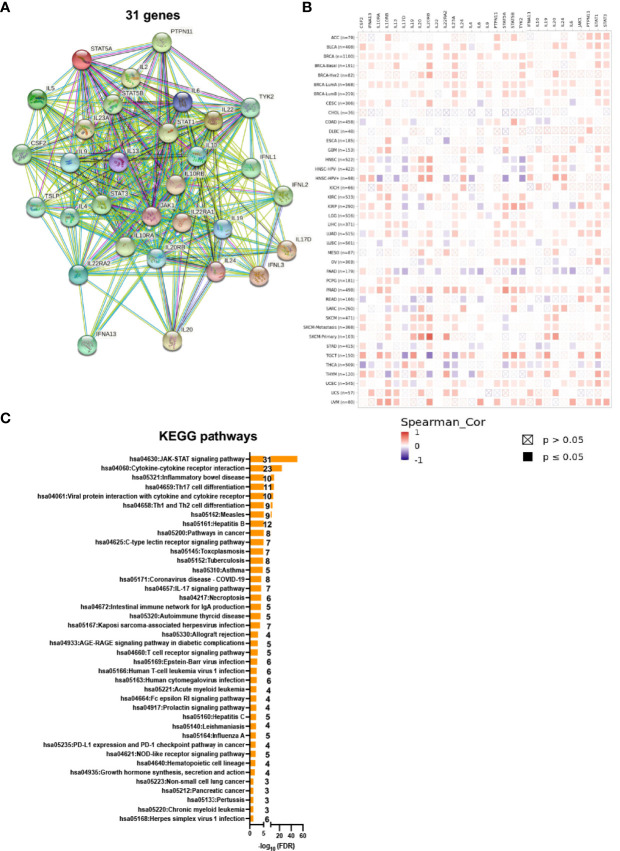
IL22RA1-associated genes are involved in the JAK-STAT signaling pathway. A total of 30 genes were related with IL22RA1 based on https://www.ndexbio.org/iquery/. **(A)** Network showing protein-protein interaction for the 31 genes. **(B)** Correlation between genes related with IL22RA1 and IL22RA1 in multiple cancers. **(C)** Function analysis of these 31 genes based on Kyoto Encyclopedia of Genes and Genomes (KEGG) pathways using DAVID. Significant pathways shown (FDR < 0.05) with gene number for each pathway.

A total of 30 genes (*STAT5B*, *STAT5A*, *IL13*, *CSF2*, *IL10RA*, *IL20RB*, *TYK2*, *IL9*, *IL23A*, *IL10RB*, *IL22RA1*, *IFNL2*, *IL22*, *IFNL1*, *IL24*, *IL19*, *IFNL3*, *IL17D*, *IL20*, *IFNA13*, *IL22RA2*, *IL6*, *IL4*, *PTPN11*, *IL2*, *IL10*, *JAK1*, *STAT1*, *STAT3*, *IL5*, and *TSLP*) were related with *IL22RA1* based on https://www.ndexbio.org/iquery/. Protein interaction network also showed a relationship between these 31 proteins **(** **Figure 2A** **)**. We further explored the correlation of *IL22RA1* and the related 30 genes in each cancer (from TIMER2). *IL22RA1* was positively correlated with *STAT1*, *STAT3*, *JAK1*, *PTPN11*, *IFNA13*, *IL24*, but negatively correlated with *IL10* in specific cancers **(** **Figure 2B** **) (** **Table S1** **)**.

To understand the function of the 31 genes, we performed KEGG pathway analysis using DAVID and found that all genes were involved in the JAK/STAT signaling pathway **(** **Figure 2C** **)**.

### IL22RA1-Related Genes Were Associated With Overall Survival Rate in Patients

**Figure 3:**
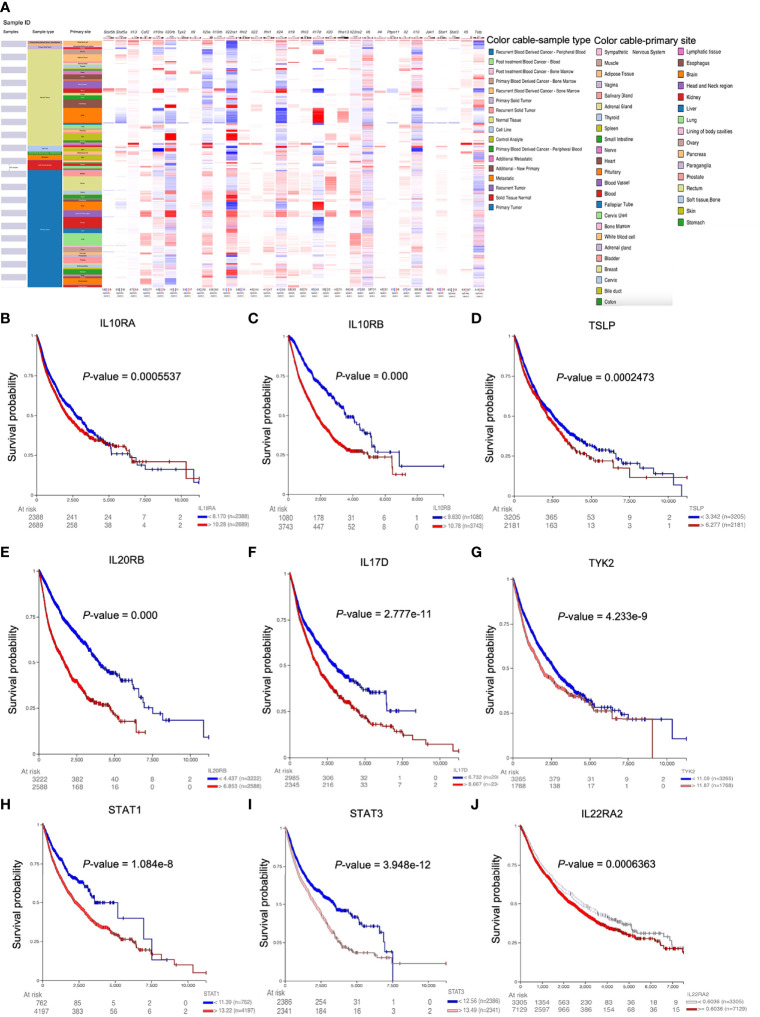
Higher expression of IL22RA1-associated genes correlates with overall survival in patients. **(A)** Heatmap shows the expression of IL22RA1 and IL22RA1-related genes in tumor and normal tissues from TCGA/TARGET/GTEx. **(B–J)** Influence of the expression of indicated genes on overall survival rate based on UCSC Xena web-based tool.

Consistent with IL22RA1, among IL22RA1-related genes, low expression of *IL10RA*, *IL10RB*, *TSLP*, *IL20RB*, *IL17D*, *TYK2*, *STAT1*, *STAT3*, and *IL22RA2* were associated with better survival in patients by pan-cancer analysis **(** **Figure 3** **)**.

### IL22RA1 Expression Was Associated With immune Cell Infiltration by TIMER2.0 Estimation

IL22 is derived from immune cells, and IL22 level can influence the expression of IL22RA1 (10). We then used TIMER2.0 to evaluate the correlation between IL22RA1 expression and infiltration level of immune cells estimated by all six algorithms across TCGA cancer types (11). As expected, the upregulated expression of IL22RA1 in 11 cancers was correlated with different immune cell infiltrates. Association with significant difference is shown in **Table 1**. The correlation between IL22RA1 expression and immune cell infiltration depends on cancer type. For example, IL22RA1 was positively correlated with CD8+ T cells (rho = 0.38) in BLCA which was opposite in STAD.

**Table 1:** Correlation between IL22RA1 and immune cells in cancers with significance.

Cancer	Infiltrates	Database	rho	*p*	adj.*p*
BLCA (n=408)	T cell CD8+	TIMER	0.375870861	8.57E-14	5.71E-12
BLCA (n=408)	T cell CD4+ Th2	XCELL	0.240040351	3.20E-06	0.00010619
BLCA (n=408)	Myeloid dendritic cell	TIMER	0.237558592	4.07E-06	7.53E-05
BLCA (n=408)	Macrophage M0	CIBERSORT	0.202692024	9.01E-05	0.00097276
BLCA (n=408)	T cell NK	XCELL	-0.22469057	1.35E-05	0.00027053
BLCA (n=408)	Macrophage M2	TIDE	-0.265003729	2.48E-07	8.77E-06
CESC (n=306)	T cell CD4+	EPIC	0.252022829	2.19E-05	0.00049351
CESC (n=306)	Myeloid dendritic cell activated	CIBERSORT-ABS	0.22993318	0.00011274	0.00135965
CHOL (n=36)	T cell CD8+ effector memory	XCELL	-0.440181078	0.00814013	0.03876253
ESCA (n=185)	T cell CD4+ memory resting	CIBERSORT	0.246807835	0.0008377	0.01034805
ESCA (n=185)	T cell regulatory (Tregs)	CIBERSORT	0.208691008	0.00493263	0.03198381
ESCA (n=185)	Macrophage M2	TIDE, CIBERSORT, QUANTISEQ	-0.202781909	0.00633201	0.02602196
ESCA (n=185)	Myeloid dendritic cell resting	CIBERSORT-ABS	-0.212632641	0.0041604	0.02496328
ESCA (n=185)	Macrophage/Monocyte	MCPCOUNTER	-0.214880679	0.0037704	0.0171822
ESCA (n=185)	Myeloid dendritic cell resting	CIBERSORT	-0.22573523	0.00231228	0.01560499
ESCA (n=185)	Cancer associated fibroblast	EPIC	-0.268325664	0.00027063	0.00188264
ESCA (n=185)	Macrophage	TIMER	-0.287547841	9.08E-05	0.00097276
ESCA (n=185)	Cancer associated fibroblast	MCPCOUNTER, XCELL, TIDE	-0.311235972	2.11E-05	0.0002414
KIRC (n=533)	Macrophage/Monocyte	MCPCOUNTER	0.394215155	1.37E-18	8.20E-16
KIRC (n=533)	Macrophage M1	QUANTISEQ	0.371131488	1.68E-16	5.03E-14
KIRC (n=533)	T cell CD4+ effector memory	XCELL	0.304993488	2.22E-11	2.33E-09
KIRC (n=533)	Macrophage M2	XCELL	0.261457064	1.21E-08	7.28E-07
KIRC (n=533)	Macrophage	EPIC, XCELL	0.238954435	2.08E-07	8.32E-06
KIRC (n=533)	T cell CD8+ effector memory	XCELL	0.202298739	1.20E-05	0.00027353
KIRC (n=533)	Myeloid dendritic cell	TIMER	0.200561735	1.43E-05	0.00020326
KIRC (n=533)	Macrophage M2	TIDE	-0.203868047	1.03E-05	0.00021213
KIRP (n=290)	Macrophage/Monocyte	MCPCOUNTER	0.430030266	4.90E-13	7.35E-11
KIRP (n=290)	T cell CD4+ (non-regulatory)	QUANTISEQ	0.306746724	5.04E-07	2.44E-05
KIRP (n=290)	NK cell	QUANTISEQ	0.305413211	5.68E-07	2.02E-05
KIRP (n=290)	Plasmacytoid dendritic cell	XCELL	-0.239781291	0.00010039	0.00124858
KIRP (n=290)	Macrophage M1	CIBERSORT	-0.249759539	4.98E-05	0.00066353
LIHC (n=371)	Macrophage M1	CIBERSORT-ABS	0.239900403	6.61E-06	0.00015655
LIHC (n=371)	T cell CD4+ memory resting	CIBERSORT-ABS	0.202900672	0.00014789	0.00245193
LUAD (n=515)	T cell CD4+	EPIC	0.260107312	4.58E-09	2.88E-07
LUAD (n=515)	Macrophage M2	TIDE	-0.201121877	6.78E-06	0.00015655
LUSC (n=501)	Macrophage1	XCELL	-0.205633542	5.96E-06	0.000149
STAD (n=415)	NK cell resting	CIBERSORT	0.238313484	2.71E-06	7.22E-05
STAD (n=415)	T cell CD4+ naive	XCELL	-0.205045951	5.78E-05	0.0012147
STAD (n=415)	Myeloid dendritic cell activated	XCELL	-0.220478008	1.48E-05	0.00020326
STAD (n=415)	T cell CD8+ central memory	XCELL	-0.221639465	1.33E-05	0.00028028
STAD (n=415)	T cell CD8+	XCELL	-0.238456104	2.67E-06	0.00010674
STAD (n=415)	Myeloid dendritic cell	QUANTISEQ, MCPCOUNTER, XCELL	-0.245808023	1.27E-06	3.52E-05
STAD (n=415)	Cancer associated fibroblast	XCELL	-0.262854121	2.08E-07	4.76E-06
UCEC (n=545)	Myeloid dendritic cell activated	CIBERSORT	0.34484618	0.00100105	0.00830034

In CHOL, CD8+ effector memory T cells were also positively correlated with IL22RA1 (rho = 0.44). It was positively correlated with CD4+ Th2 cells in BLCA (rho = 0.24), as well as CD4+ T cells and regulatory T cells (Tregs) in CESC (rho = 0.25), CD4+ effector memory T cells in KIRC (rho = 0.30), CD4+ T cells in LUAD (rho = 0.26). In addition to T cells, IL22RA1 was positively correlated with myeloid dendritic cells (rho = 0.23) and macrophage M0 in BLCA (rho = 0.20) as well as macrophage/monocyte in KIRP (rho = 0.43) and KIRC (rho = 0.39) **(** **Table 1** **)**.

### Mutation Incidence of IL22RA1 in Cancer

**Figure 4:**
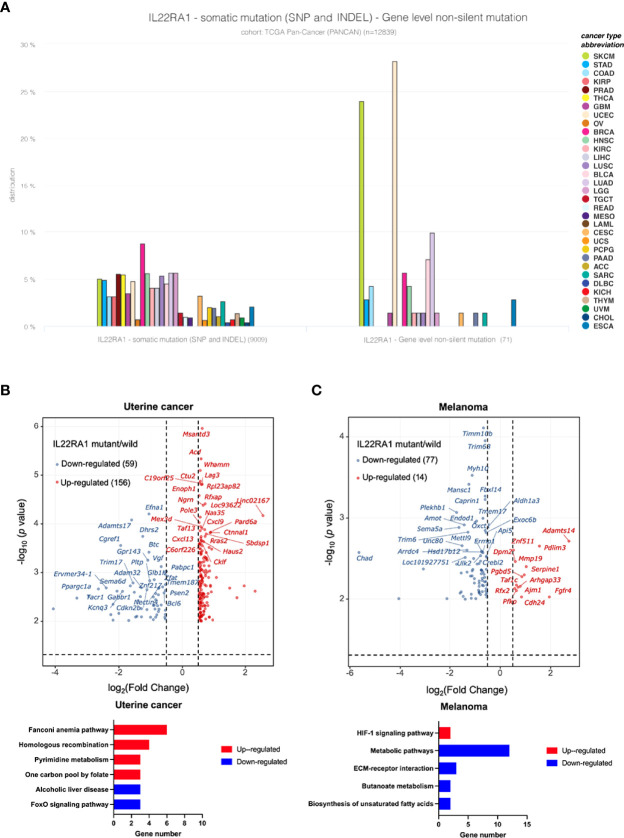
IL22RA1 mutation affects metabolic pathways in uterine cancer and melanoma. **(A)** Incidence of IL22RA1 mutation in different cancer types shows both UCEC and SKCM are major cancer types with higher mutation rate. **(B)** Volcano plot displaying the differentially expressed genes (|fold change|>1.5 and adjusted *p* < 0.05) after IL22RA1 mutation in uterine cancer (upper), and KEGG pathways for the upregulated and downregulated genes. **(C)** Volcano plot displaying differentially expressed genes (|fold change| >1.5 and adjusted *p* < 0.05) after IL22RA1 mutation in melanoma (upper), and KEGG pathways for the upregulated and downregulated genes.

As both genomic and transcriptomic changes are important signatures for cancer development, and gene mutation can alter the expression of certain genes. We further wanted to investigate whether mutation of IL22RA1 can change gene expression, and what are the functions of the affected genes. The non-silent mutation among diverse cancer types is mainly distributed in uterine cancer (>25%) and melanoma (> 20%) **(** **Figure 4A** **)**.

Based on mutant (n = 19) versus wild-type (n = 507) in uterine cancer, IL22RA1 mutation upregulated 156 genes, which involved in DNA repair (fanconi anemia pathway [*SLX4*, *RAD51*, *EME1*, *FANCA*, *BRCA1*], homologous recombination [*RAD51*, *EME1*, *RBBP8*, *BRCA1*]), and one carbon pool by folate [*DHFR*, *MTHFD2L*, *TYMS*], whereas downregulated 59 genes involving alcoholic liver disease (*C3*, *PRKAG2*, *PPARGC1A*) and the FoxO signaling pathway (*CDKN2B*, *BCL6*, *PRKAG2*) **(** **Figure 4B** **)**. Gene encoding CCND1 was also upregulated in IL22RA1 mutant compared with wild-type. *CCND1* is dysregulated in many cancers, indicating mutation of IL22RA1 contributes to cancer development **(** **Figure 4B** **)**.

In melanoma, a total of 14 genes were upregulated, whereas 77 genes were downregulated when comparing IL22RA1 mutated and wild-type patient cohorts. Upregulated *SERPINE1* and *PFKP* by IL22RA1 mutation were involved in the HIF-1 signaling pathway. However, genes in biosynthesis of unsaturated fatty acids (*HSD17B12*, *FADS1*), butanoate metabolism (*ALDH5A1*, *OXCT1*), and other metabolic pathways (*PDHX*, *ALDH1A3*, *HSD3B2*, *PDE3B*, *MBOAT1*, *CTPS2*, *CMBL*, *B4GALT6*), as well as ECM-receptor interaction (*SV2B*, *ITGB3*, *CHAD*) were downregulated by IL22RA1 mutation **(** **Figure 4C** **)**.

### Validation of IL22RA1 Expression in Uterine Cancer From Animals and Humans

**Figure 5:**
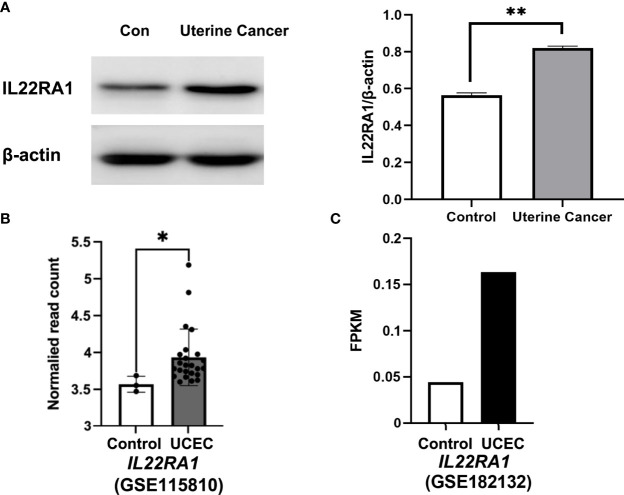
IL22RA1 expression in uterine cancer at protein and transcript level. **(A)** Representative Western blot results for IL22RA1 in uterine cancers from dogs (left panel). Results are presented as the ratio of the IL22RA1 band intensity to the intensity of the β-actin band (n = 3, right panel). **(B, C)** RNA seq data showing the transcript level of IL22RA1 in UCEC tissues and normal control tissues using GSE115810 and GSE182132 cohorts from GEO database. *p < 0.05, **p < 0.01.

The protein level of IL22RA1 was increased in uterine cancer compared with normal control tissues from dogs (*p* < 0.01; **Figure 5A**). The transcript level of IL22RA1 in UCEC was confirmed using human datasets from GEO database. Compared with the control, *IL22RA1* transcript was higher in UCEC tissues (GSE115810; *p* < 0.05) (**Figure 5B**). Consistently, the transcript level of IL22RA1 in UCEC case was 3-fold change higher than the control in humans (GSE182132) (**Figure 5C**).

## Discussion

Cytokine and cytokine receptor interaction is the pathway related with inflammation and cancer therapy (12). IL22 is multifunctional and involved in various diseases including cancer. We previously investigated the role of IL22 signaling in response to infection, and found that IL22 can be upregulated by probiotics and provide a protective effect against pathogens at barrier surfaces (13–15). IL22 is produced by immune cells (CD4+ helper T, CD8+ cytotoxic T, NKT, γδ T, and innate lymphoid cells) in numerous tissues. However, the expression of IL22 in systemic organs is usually low. IL22 acts by binding its receptor, a heterodimeric receptor complex including IL22RA1 and IL10RB. It has been confirmed that the IL22/IL22RA1 pathway modulates the expression of many genes involved in survival, differentiation, and remodeling (13, 16). In this study, we demonstrated the role of receptor of IL22 (IL22RA1) in cancers by studying the crosstalk between IL22RA1 and immune system.

IL22RA1 expression is abundant in the pancreas, gastrointestinal tissues and liver at transcriptional level. At single cell level, IL22RA1 is mainly expressed on non-immune cells, especially epithelial cells. Pan-cancer analysis shows that IL22RA1 is highly expressed in 11 types of tumors including uterine cancer. Consistently, we identified that the expression of IL22RA1 in uterine cancer was increased significantly at both transcript and protein level. Remarkably, upregulation of IL22RA1 is associated with decreased overall survival in patients. Consistent with other studies (4, 17), it has been found that IL22 and its receptor IL22RA1 promote tumorigenicity and contribute to pathogenesis.

We further performed correlation analysis between IL22RA1 and tumor infiltrating immune cells in different types of cancer, as IL22 is produced mainly by immune cells. It has been found that IL22-producing CD4+ T cells promote colorectal cancer stemness which enhance tumorigenesis (18). IL22RA1 was negatively associated with monocytes but positively correlated with macrophages M1 in lung adenocarcinoma (19). IL22 can increase the mRNA expression of IL22RA1 by promoting binding of STAT3 directly to the promoter region of IL22RA1 (4, 10). Our study shows that correlation between IL22RA1 expression and different immune cells infiltration depends on the type of cancer. In consistency, the principal cellular source of IL22 is various between tissues (10). It suggests that regulation of IL22RA1 and IL22 in different tumors is involved in targeting different immune cell populations. However, the relationship between IL22RA1 and various types of IL22-producing cells in specific tumor tissues still needs to be confirmed in the future.

IL22RA1 together with the other 30 genes interact with each other and are involved in the Janus kinase/signal transducers and activators of transcription (JAK/STAT) pathway. The JAK/STAT signaling pathway directly regulates the crosstalk between transmembrane receptors to the nucleus (20). Through binding to IL22RA1 and IL10R2, IL22 can activate JAK1/TYK2 kinases and downstream phosphorylation of STAT proteins, in particular STAT3 (21). JAK activation usually stimulates cell proliferation, differentiation, cell migration and apoptosis. However, activation of the JAK/STAT signaling pathway contributes to cancer progression (22, 23). IL22 promoted malignant transformation of mesenchymal stem cells in rats through IL22RA1/STAT3 signaling (7). In addition, STAT1 and STAT3 are involved in PD-L1 expression and PD-1 checkpoint pathway in cancer by pathway analysis. We have found that high level of STAT3 and STAT1 transcripts was associated with poor survival in patients through pan-cancer analysis.

Consistent with IL22RA1, higher expression of IL10RB, IL10RA and IL20RB in tumor tissues was associated with worse survival in patients. IL22R1 is expressed only by nonhematopoietic cells, whereas IL10RB (IL10R2) is ubiquitously expressed by all tissues and cells (24). Overexpression of IL10R2 contributes to colorectal carcinogenesis by IL22/STAT3 signaling (24). IL20RB has been proposed as a prognostic and therapeutic biomarker in clear cell renal cell carcinoma (25).

Overexpressed thymic stromal lymphopoietin (TSLP) and tyrosine kinase 2 (TYK2) was also associated with worse survival through pan-cancer analysis. TSLP is an epithelial-cell–derived cytokine that contributing to inflammation driven by Th2 cells (26). TSLP also has an important role in cancer, and the pro- or anti-tumor effect is dependent on the type of tumor (27, 28). TYK2 belongs to subfamily of JAKs involved in cytokine signaling including the IL22/IL22RA1 signaling. TYK2 acts as an oncogene, and its overexpression has been detected in many types of cancer (29–31). Taken together, our analysis indicates that IL22RA1 contributes to tumor progression through activating the JAK/STAT signaling pathway.

Gene alterations lead to cancer initiation and driving progression (32). Silent mutations can affect gene expression which may relate to tumorigenesis and cancer cell fitness (33). It is worth to note that the non-silent mutation rate of IL22RA1 is high in UCEC and SKCM compared with the other types of cancer. IL22RA1 mutation in uterine cancer upregulated genes in the fanconi anemia pathway and homologous recombination which were involved in DNA damage and repair. Upregulation of the fanconi anemia pathway components such as RAD51 and BRCA1/2, leads to homologous recombination restoration and drug resistance (34, 35).

In melanoma, IL22RA1 mutation upregulated the expression of genes involved in the hypoxia-inducible factor-1alpha (HIF-1α) signaling pathway, whereas the metabolic pathways including biosynthesis of unsaturated fatty acids and butanoate metabolism were downregulated. Hypoxia, as a major feature of solid tumors, activates HIF-1α that enhances tumor growth and leads to melanoma progression by regulating the expression of genes involved in angiogenesis, metabolism, cell proliferation, and metastasis (36).

In conclusion, IL22RA1 together with genes involved in the JAK/STAT pathway contribute to cancer development. Immune cell infiltration level in tumor tissues is associated with IL22RA1 expression. IL22RA1 mutation in uterine cancer and melanoma induced overexpression of genes that are favorable in tumor progression. These findings indicate that IL22RA1 may act as a contributor to tumorigenesis with a direct and indirect role in the process. However, more detailed mechanism in specific cancer still needs to be investigated.

## Data Availability Statement

The original contributions presented in the study are included in the article/**Supplementary Material**. Further inquiries can be directed to the corresponding author.

## Ethics Statement

The animal study was reviewed and approved by Ethical Committee of Northeast Agricultural University. Written informed consent was obtained from the owners for the participation of their animals in this study.

## Author Contributions

Conceptualization, GY; Data analysis and writing, SZ and GY; All authors have read and agreed to the published version of the manuscript.

## Funding

This work was supported by the National Natural Science Foundation of China Grant (Grant No. 32002351) and International Postdoctoral Exchange Fellowship Program 2019 of China Postdoctoral Council (No. 40 Document of OCPC, 2019).

## Conflict of Interest

The authors declare that the research was conducted in the absence of any commercial or financial relationships that could be construed as a potential conflict of interest.

## Publisher’s Note

All claims expressed in this article are solely those of the authors and do not necessarily represent those of their affiliated organizations, or those of the publisher, the editors and the reviewers. Any product that may be evaluated in this article, or claim that may be made by its manufacturer, is not guaranteed or endorsed by the publisher.
