# Supplementary material for: IL22RA1/JAK/STAT Signaling Acts As a Cancer Target Through Pan-Cancer Analysis
Source: Front Immunol. 2022 Jul 8;13:915246. doi: 10.3389/fimmu.2022.915246 (PMC9304570; doi:10.3389/fimmu.2022.915246)
Supplement: Supplementary file 1 [file Table_1.docx]

| **Cancer** | **Infiltrates** | **rho** | ***p*** | **adj.*p* (<0.05)** |
| --- | --- | --- | --- | --- |
| KIRC (n=533) | TYK2 | 0.26285666 | 1.01E-08 | 2.75E-07 |
| KIRP (n=290) | TYK2 | 0.36458512 | 1.57E-09 | 5.66E-08 |
| ESCA (n=185) | TSLP | -0.2343025 | 0.00154665 | 0.00902215 |
| KIRP (n=290) | TSLP | 0.20258386 | 0.00106724 | 0.00731776 |
| CESC (n=306) | TSLP | 0.22211896 | 0.00019397 | 0.00167807 |
| LUSC (n=501) | STAT5B | 0.2052354 | 6.22E-06 | 8.35E-05 |
| KIRP (n=290) | STAT5B | 0.44659479 | 4.73E-14 | 3.75E-12 |
| KIRC (n=533) | STAT5A | 0.22931927 | 6.46E-07 | 1.14E-05 |
| UCEC (n=545) | STAT5A | 0.23429393 | 5.13E-05 | 0.00044123 |
| KIRP (n=290) | STAT5A | 0.31498505 | 2.38E-07 | 4.71E-06 |
| KIRP (n=290) | STAT3 | 0.20659298 | 0.00084278 | 0.00616322 |
| LIHC (n=371) | STAT1 | 0.22733671 | 2.02E-05 | 0.00024531 |
| BLCA (n=408) | STAT1 | 0.28926598 | 1.59E-08 | 4.45E-07 |
| UCEC (n=545) | STAT1 | 0.36467332 | 1.21E-10 | 8.44E-09 |
| BLCA (n=408) | PTPN11 | 0.2091987 | 5.25E-05 | 0.0004469 |
| ESCA (n=185) | JAK1 | 0.21535263 | 0.00369283 | 0.01879988 |
| KIRP (n=290) | JAK1 | 0.31905917 | 1.63E-07 | 3.51E-06 |
| ESCA (n=185) | IL22RA2 | -0.3040938 | 3.32E-05 | 0.0003121 |
| LUSC (n=501) | IL20RB | 0.22806931 | 4.79E-07 | 8.82E-06 |
| STAD (n=415) | IL20RB | 0.2320729 | 4.98E-06 | 6.92E-05 |
| BLCA (n=408) | IL20RB | 0.23649806 | 4.50E-06 | 6.37E-05 |
| KIRC (n=533) | IL20RB | 0.32807877 | 4.97E-13 | 3.03E-11 |
| CESC (n=306) | IL20RB | 0.35270031 | 1.55E-09 | 5.66E-08 |
| KIRP (n=290) | IL20 | -0.2130563 | 0.00057061 | 0.00353064 |
| BLCA (n=408) | IL20 | 0.23749394 | 4.10E-06 | 6.12E-05 |
| UCEC (n=545) | IL2 | -0.2386727 | 3.66E-05 | 0.00041027 |
| KIRP (n=290) | IL17D | -0.2275414 | 0.00022819 | 0.00166709 |
| LIHC (n=371) | IL17D | 0.2241307 | 2.65E-05 | 0.00026582 |
| UCEC (n=545) | IL17D | 0.23438661 | 5.09E-05 | 0.00044123 |
| CESC (n=306) | IL10RB | 0.22720152 | 0.00013658 | 0.00107097 |
| LIHC (n=371) | IL10RB | 0.23671424 | 8.82E-06 | 0.00011273 |
| KIRP (n=290) | IL10RB | 0.28141086 | 4.40E-06 | 6.37E-05 |
| ESCA (n=185) | IL10RB | 0.31100799 | 2.14E-05 | 0.00023577 |
| UCEC (n=545) | IL10RB | 0.31562879 | 3.36E-08 | 8.42E-07 |
| KIRC (n=533) | IL10RB | 0.33932149 | 6.93E-14 | 4.58E-12 |
| STAD (n=415) | IL10RA | -0.2055828 | 5.53E-05 | 0.00046557 |
| KIRC (n=533) | IL10RA | 0.21476252 | 3.27E-06 | 5.08E-05 |
| BLCA (n=408) | CSF2 | 0.25179436 | 9.94E-07 | 1.61E-05 |

**Table S1.** Correlation between IL22RA1 and IL22RA1-related genes in cancers with significance
